# Towards Urbanome the genome of the city to enhance the form and function of future cities

**DOI:** 10.1038/s41467-019-11972-6

**Published:** 2019-09-05

**Authors:** Lidia Morawska, Wendy Miller, Matt Riley, Sotiris Vardoulakis, Yong-Guan Zhu, Guy B. Marks, Prachi Garnawat, Prashant Kumar, Marie Thynell

**Affiliations:** 1 Queensland University of Technology, International Laboratory for Air Quality & Health (ILAQH), Brisbane, QLD Australia; 2 Queensland University of Technology, Science and Engineering Faculty, Brisbane, QLD Australia; 3 Queensland University of Technology, Institute of Health Biomedical Innovation (IHBI), Brisbane, QLD Australia; 4 NSW Office of Environment and Heritage (NSW OEH), Climate and Atmospheric Science, Sydney, Australia; 5 Institute of Occupational Medicine (IOM), Edinburgh, UK; 6 Key Lab of Urban Environment and Health, Institute of Urban Environment, Chinese Academy of Science, Xiamen, China; 7 South Western Sydney Clinical School, University of New South Wales, Sydney, NSW Australia; 8 Woolcock Institute for Medical Research, University of Sydney, Sydney, NSW Australia; 9 RMIT University, Melbourne, Australia; 10 Global Centre for Clean Air Research (GCARE), Department of Civil and Environmental Engineering, Faculty of Engineering and Physical Sciences, University of Surrey, Guildford, GU2 7XH Surrey UK; 11 University of Gothenburg, School of Global Studies, Göteborg, Sweden

**Keywords:** Environmental impact, Environmental social sciences, Sustainability

## Abstract

The health of the city depends on how well all the elements of this system are interconnected and operating in harmony. Here the authors introduced the concept of urbanome which is analogous to the human genome that can be used to characterise the form and functioning of cities.

Over the past one hundred years or so a significant shift has occurred in human population distribution worldwide: from 13% of people living in cities in 1900 to 55% 2018, with a further expected increase to 68% by 2050^1,2^. This incredibly fast change, taking place within the lifespan of two or three human generations, is in fact a blink in the history of humanity. This is a shift from small rural communities living close to nature, to enormous man-made systems that are largely disconnected from nature. Urbanisation can have both positive and negative impacts^3^, but the complexity of urban systems makes it difficult to determine to what extent our cities are functioning to provide for the many needs of modern communities in high, middle and low-income countries. What framework can encompass the complexity of the system to help us understand it and manage our cities to the greatest extent possible? To do this, we propose to construct the urbanome, a framework to characterise cities and people living in them in a holistic manner.

## The definition of Urbanome

We define the term urbanome as a complete set of data describing the physical, social, operational and structural characteristics of a city, and its impacts, placed on a common platform, where it could be analysed to diagnose problems, identify best solutions to them and test future scenarios, policies and interventions using a holistic systems approach.

## Different facts and aspects of the concept of the Urbanome

We propose that urbanome is analogous to the human genome, which facilitates understanding of human biology^4^. Similar to the genome, the urbanome would be the holistic mapping (“sequencing”) of the key characteristics of a city and its residents. This can be done at different spatial and temporal scales depending on the urban settings, local needs and information available. In principle, it could include spatially and temporally resolved data that could be used to assess any aspect of a city, make comparisons between cities or between neighbourhoods within the same city, and inform solutions. Although the genome is the main framework, there are other ‘omics’ which are relevant for the development of the urbanome, including biome^5^, exposome^6^, or pollutome^7^. Tracing the formulation and evolution of the existing ‘omics’ it is evident that none of these frameworks came to be in their final form; instead, they all evolved over time benefiting from advances in science and technology, with the many elements of their utility being apparent only with time. The best example is the genome already completed as a database for humans, but with new discoveries regularly being announced of how this ‘map’ can be used to diagnose and alleviate the existing and future potential health problems^8–10^.

## Scientific approaches to development of the Urbanome

The urbanome concept is not only inspired by the obvious interactions and interdependencies between the natural, human and built environments, but also by the methods and approaches that have been developed by scientists working in the natural, health and built environment disciplines. An operational urbanome, a uniform and transparent framework that could be used across cities, could be developed through a transdisciplinary approach that borrows, adapts and shares methodologies from these usually discrete sciences. For example, the scale at which a system can be understood is seen by comparing the genome (the very small) with the biome (the very large) – yet both seek to characterise the fundamentals that make the system (an organism or a large ecological area) function, how they are linked, to what extent they can explain or identify possible dysfunction, and how they a similar to, or different from, other systems. How things grow and function can also be studied at different scales and contexts, as demonstrated for organisms through the study of metabolism and in cities through the study of urban metabolism. The identification of key health determinants and their distribution is studied at a population scale through epidemiology, but the methods used can also be applied to urban environments, for example through energy epidemiology.

## The importance of the concept

Learning from the application of other ‘omics’ to their respective fields, the urbanome will enable the characterisation and inter comparisons of cites; the characterisation of trends within a city; diagnosing existing and/or predicting future urban ills; and identifying optimal solutions. A transdisciplinary and multidisciplinary approach to the development of an urbanome framework will enable such a framework to understand and characterise the fundamental base units that comprise a city, to characterise similarities and differences between cities, to determine how cities grow and function, and to manage key health determinants and their distribution.

Being able to compare and learn from the outcomes of other cities will undoubtedly accelerate progress of the cities still developing, similarly to the gene expression, which identify individual group of genes present and thus signalling potential problems. This does help in identifying and eliminating genetic causes of disease, to make a person healthier now and avoid problems in the future. Therefore, using the urbanome will help reducing the burden on society from living in inadequate cities (e.g. health burden), and in applying interventions with positive impacts on an individual, society and future generations.

Based on the history of the genome or biome, we know that much work will be needed to fully develop the urbanome as a working product. This will require much greater efforts carried out by much larger transdisciplinary teams, than the formulation of the concept by the group of the co-authors of this work.

The development of the urbanome could be much accelerated compared to the developments in the past of other ‘omics’ due to the unprecedented progress in Big Data acquisition, storage and manipulation. There is an enormous potential in harnessing Big Data (e.g. health, environmental, socioeconomic), and metrics and indicators that can be used for decision making in an operational way. One of the foci of building the urbanome will be the discussion on how the data could be brought together, analysed and visualised. Further, there will be a need to identify the core data that indicates current levels of urban resilience and how resilience changes over time (improves, decreases) in response to different stressors. This will require indicators at the level of neighbourhoods, because resilience varies amongst sub-groups/neighbourhoods and from one part of a city to another (e.g.^11^). The spatiotemporal resolution of data forming the urbanome will be a very important aspect since aggregate data and indicators often mask exposure to pollution, unhealthy conditions or socioeconomic gradients within cities. A fundamental issue will be the discussion on data quality and availability (information asymmetry), particularly for low- and middle-income countries, as well as data privacy and security.

Next, consideration will need to be given to the statistical and analytical tools necessary for identifying the presence and diagnosing the causes of ‘urban health’ problems. Of course, there are many tools available for spatial and time-series analysis. Are they adequate, or would new tools be needed?

The ultimate product will have the data categorized into different groups (environmental, socioeconomic, etc.), and create a platform for data integration and data science-based decision making. Further, environmental and health data could be used to identify population sub-groups, households or even individuals that are vulnerable to urban air pollution, noise or heat (e.g. MEDMI platform^12^). Socioeconomic and health data could be used to detect health inequities at different geographical scales (e.g.,^13^).

Although the availability and quality of the data are issues in certain urban settings (particularly in low income cities), access to current information technology, artificial intelligence and data mining techniques enable the development of large multi-dimensional databases that can support the urbanome concept today and into the future. Citizen science also offers opportunities for data collection through widespread use of mobile technologies, GPS and low-cost sensors (e.g. CITI-SENSE project^14^). The urbanome would help to identify different urban indicators or composite indices that may integrate specific datasets and serve a variety of city governance purposes, e.g. traffic management, health care, environmental protection.
